# Medical Society, of the District of Columbia

**Published:** 1878-02

**Authors:** 


### *Medical Society of the District of Columbia.*

The Medical Society of the District of Columbia assembled Thursday night, Dec. 20, at Marini's Hall, which was well filled The occasion was to celebrate the sixtieth anniversary of the society.

Dr. Toner, as chairman of the committee of arrangements, in announcing the programme for the evening, made, in substance, the following remarks: The Medical Society of the District of Columbia was founded September 26, 1817, and this meeting therefore represents the 60th anniversary—a period of time which represents two generations of human life. Our first charter was obtained from congress in March, 1819. The District then embraced the original ten miles square, with the three cities, Washington, Georgetown and Alexandria, the whole population being but little over 30,000. There were twenty-one physicians named in this charter, all of whom are now deceased, and they included nearly all the medical men then practicing in the territory named. I use the term our first charter, because we are now acting under a revised and amended act of incorporation granted July 7, 1838. In our present charter there are named twenty-two physicians, but the nearly forty years that have supervened since that time, have removed from earth all but seven of our honored and worthy representatives. The names of those living are Doctors J. B. Blake, Joseph Borrows, H. F. Condict, J. C. Hall, Benjamin King, Harvey Lindsly, and Noble Young. Of these, two—Drs. J. B. Blake and J. C. Hall—have been physicians for over fifty years; and Drs. Joseph Borrows, Harvey Lindsly and Noble Young, will have been physicians fifty years next March. All these physicians are as familiar to the citizens of Washington as household words, and have been particularly blessed with length of days and professional usefulness, beyond the average allotted to medical men in the United States; the average life of physicians being but about 58 years. The average term of professional life in America is 32½ years; and the average age at which physicians commence to practice, is between 25 and 26 years. From the formation of our society in 1817 to the present, there have been about 450 names enrolled as licentiates of this society. Of these about 180 survive, and are more or less actively engaged in practice. Without further prefatory remarks, I proceed to the discharge of the agreeable duty assigned to me of presenting to you an old and highly esteemed member, who has consented to be the orator of the evening—Dr. A. Y. P. Garnett.

Dr. Garnett opened his able and interesting address with a reference to the rapid progress of the science of medicine, due mainly to the organized efforts of the profession, particularly such associations as this. He gave a rapid glance at the history of medical practice and schools of medicine, ending with a highly interesting account of the straits to which the Confederate surgeons and physicians were reduced during the war by the enforcement of the Federal blockade, which partially deprived them of their supplies. The physicians cut off from their accustomed vegetable drugs, substituted indigenous plants with great success. He complimented them for successfully battling with the emergency, and also complimented the surgeons for devising new methods of practice, made necessary by the blockade, particularly Prof. Campbell, for a valuable discovery which he described. He closed with a tribute to the present high character of the profession generally, and the improvements in the science of medicine due to this.

